# Kreasote in Gangrene

**Published:** 1860-10

**Authors:** 


### ARTICLE XXIII.

### *Kreasote in Gangrene.*

Dr. Parke, an American physician who served in the Russian Hospital in the Crimean war, in speaking of the treatment of gangrene in sores and stumps, says :—

I cannot speak too highly of kreasote in this disease. I have seen gangrenous stumps looking perfectly healthy in forty eight hours, and sometimes in twenty-four, after the application of kreasote in the following manner. Where the gangrene had affected the stump to the extent of half an inch or less, I have taken the swab and applied the pure kreasote all over the diseased parts, and in the regu-ular cavities, and then covered the parts with a cloth smeared over with liniment, composed of one drachm kreasote to Oj of oil. As I said, frequently in twenty-four hours, the whole gangrenous mass would come off on the removal of the dressing. The protruding bone was never cut off, but left until it became partially absorbed within the healthy granular mass, when it was taken hold of with forceps, twisted off, and extracted. The time necessary for its absorption generally amounted to several weeks after the stump had taken on a healthy action. In conclusion, let me say, our patients had a good diet, being served with plenty of soup, good fresh meat, rye and buckwheat bread (which they preferred) and occasionally good light wheat bread, *vodka* (rye whiskey) twice a day, and sometimes wine."—*American Journal of Medical Science.*

